# Identification of alkaline pH optimum of human glucokinase because of ATP-mediated bias correction in outcomes of enzyme assays

**DOI:** 10.1038/s41598-019-47883-1

**Published:** 2019-08-06

**Authors:** Daniela Šimčíková, Petr Heneberg

**Affiliations:** Charles University, Third Faculty of Medicine, Prague, Czech Republic

**Keywords:** Kinases, Cancer metabolism

## Abstract

Adenosine triphosphate (ATP) is a crucial substrate and energy source commonly used in enzyme reactions. However, we demonstrated that the addition of this acidic compound to enzyme assay buffers can serve as a source of unnoticed pH changes. Even relatively low concentrations of ATP (up to 5 mM) shifted pH of reaction mixtures to acidic values. For example, Tris buffer lost buffering capacity at pH 7.46 by adding ATP at a concentration higher than 2 mM. In addition to the buffering capacity, the pH shifts differed with respect to the buffer concentration. High ATP concentrations are commonly used in hexokinase assays. We demonstrated how the presence of ATP affects pH of widely used enzyme assay buffers and inversely affected *K*_*M*_ of human hexokinase 2 and *S*_*0*.*5*_ of human glucokinase. The pH optimum of human glucokinase was never reported before. We found that previously reported optimum of mammalian glucokinase was incorrect, affected by the ATP-induced pH shifts. The pH optimum of human glucokinase is at pH 8.5–8.7. Suggested is the full disclosure of reaction conditions, including the measurement of pH of the whole reaction mixtures instead of measuring pH prior to the addition of all the components.

## Introduction

Enzyme assays are an integral part of research, since enzymes are fundamental for maintaining the life functions of organisms. In the course of the development of enzyme assays, we must overcome many obstacles, since every enzyme represents its own individuality, which manifests by different requirements of storage, expression and purification processes, pH and temperature stability. Therefore, an adequate and well-defined environment for every enzyme is needed in order to accurately interpret outcomes of individual enzyme assays.

Many enzyme assays possess a high potential of biased outcomes caused by an inaccurately defined pH of the reaction. The well-defined pH of the reaction depends on both a reaction buffer and other components that are necessary for the reaction to proceed^1^. In this regard, the addition of an acidic substance, such as adenosine triphosphate (ATP), can serve as a source of unnoticed pH changes in enzymatic reactions. For example, high ATP concentrations are characteristic of hexokinase (HK) assays, where they have the potential to affect the Michaelis constant *K*_*M*_ of HKs as well as the *S*_*0*.*5*_ of glucokinase (GCK). Both constants, *K*_*M*_ and *S*_*0*.*5*_, define the values of the substrate concentration at which the rate of reaction is half of the limiting rate. Both enzymes have been subjected to pharmacological studies, in which interpretations of the substrate or the inhibitor/activator affinity and imitation of physiological conditions played a prominent role^2–4^. Surprisingly, many studies on HKs and other enzymes provide little information regarding the use of ATP in their reaction mixtures, or they often disclose pH of the buffer that has been measured only before the subsequent addition of other components, including ATP (Tables S1, S2). Moreover, the pH optimum of human GCK was never determined experimentally and was only inferred from seminal studies by Salas *et al*.^5^ that used rabbit GCK instead of its human ortholog (Table S1).

HKs appear to be sensitive to pH-induced changes. Available HK structures suggest that their catalytic domains possess deep crevice between the large and small sub-domains. Glucose binds to the bottom of this crevice using hydrogen bonds to both the subdomains and their connecting region. The interface between the two subdomains is rich in acidic residues and, therefore, susceptible to pH-mediated regulation^6^. Only relatively few studies on pH kinetics of HKs were previously published; particularly scarce are data on the impact of pH on enzyme activity due to protonation/deprotonation of active site residues. In this regard, the study of calorimetric profiles of yeast HK A revealed a single thermal transition in the acidic pH and two independent transitions in the alkaline pH^6^. One of the transitions at the alkaline pH corresponds to the small subdomain and the second transition at the alkaline pH corresponds to the large subdomain. The ionization state of the acidic residues at the active site likely regulates domain movements, induce the cleft closure and therefore cause the inaccessibility of active site to glucose^6^. In bovine brain HK (i.e., HK1), the −log *V*_1_ and −log *V*_1_/*K*_*M*_ profiles displayed slopes of −1 when testing their pH kinetics with glucose and Mg ATP as substrate^7^. This is consistent with the protonation of a single group on the enzyme. Two ionizable groups were suggested to be involved in the reaction, one employed in the ATP binding and catalysis, and another playing a role in glucose binding^7^.

In the present study, we focus on enzyme assays, in which the outcomes are sensitive to the pH of the reaction. As a proof of principle, we investigated HK assays, in which the addition of ATP can serve as a source of unnoticed pH changes in enzymatic reactions. We showed how ATP concentration affects pH of different buffers and demonstrated the influence of pH on changes to the *K*_*M*_ values of human HK2 as well as the *S*_*0*.*5*_ values of human GCK.

## Methods

To test the buffering capacity of commonly used enzyme assay buffers according to changing ATP concentrations, we prepared the reaction mixtures as follows: 1 mL of the respective buffer; 0.4 mL of the GST-GCK elution buffer, with or without the tested enzyme; 0.1 M ATP in various volumes; and dH_2_O added to adjust the total volume to 2 mL. The composition of the elution buffer was as follows: 2.6 mM NADP, 0.1 mL 1 M glucose, 0.2 mL 50 mM Tris, 200 mM KCl, and 5 mM DTT; pH adjusted to 8.0. We kept all solutions at 23 °C, except for ATP and NADP, which were kept on ice. In some cases, we observed the shift in pH towards more acidic values after the addition of NADP. The amount of NADP was constant in all mixtures; therefore, the observed changes in pH were caused only by changing ATP concentration. The ATP solution was added to the buffers in a form of a 100 mM aqueous solution that was prepared directly from the ATP disodium salt hydrate powder, without any adjustment of its pH and without the addition of any salts. ATP was always added shortly before the experiments to avoid any potential issues with its stability. All chemicals were purchased from Sigma-Aldrich (St. Louis, MO).

We prepared GST-GCK as described previously^8,9^ by a one-step purification using GSTrap HP (GE Healthcare, Chicago, IL). In the case of HK2, we introduced the insert encoding HK2 into pET-28a(+) and expressed HK2 in BL21(DE3)pLysS *E*. *coli*. We induced HK2 expression by the addition of 1 mM IPTG and subsequently cultivated the cells for 16 h at 22 °C. Afterwards, we purified HK2 using HisTrap HP (GE Healthcare, Chicago, IL). We measured the pH optimum of the purified GST-GCK using a coupled reaction with glucose-6-phosphate dehydrogenase as described previously^10^. We conducted measurements at 1 mM ATP, 50 mM glucose, 100 mM Tris, for pH range of 7.5–8.8 or 100 mM glycine for pH range of 8.6–10.3. We measured HK2 and GST-GCK activity using a coupled reaction with glucose-6-phosphate dehydrogenase as described previously^10,11^. In the case of HK2, we measured enzymatic activity in the range of 0–2 mM glucose, unlike GST-GCK, in which the activity we measured was in the range of 0–150 mM. We prepared all the buffers and measured the enzyme kinetics at 23 °C, thereby excluding effects of temperature on pH of the solutions used.

We performed all measurements in three or more independent experiments, each performed in triplicate. We analyzed the obtained curves by nonlinear regression using SigmaPlot 12.0.

## Results and Discussion

**Figure 1:**
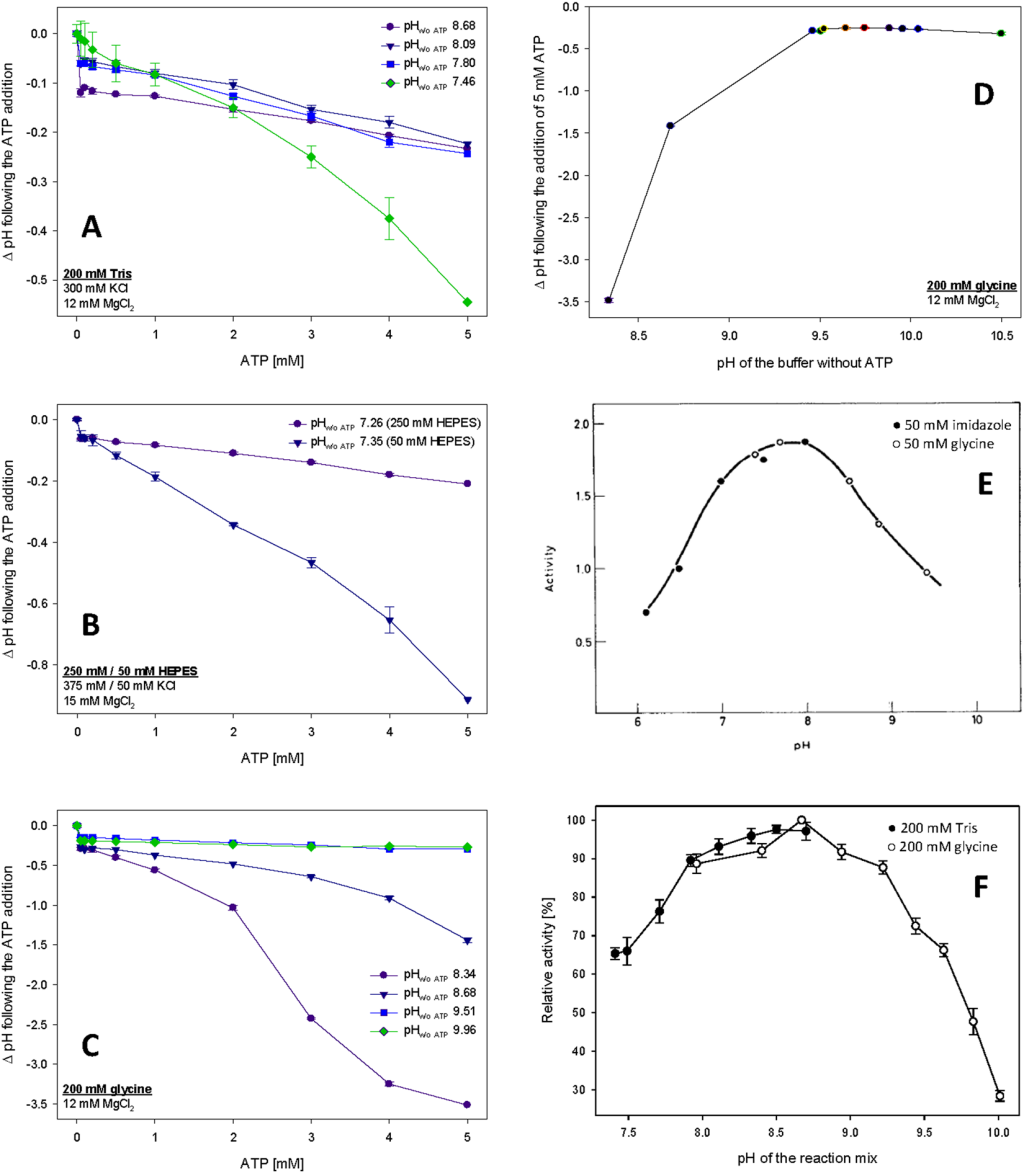
ATP addition affects pH of enzyme assay buffers and affects the measurement of the pH optimum as demonstrated in the example of GCK. (**A**) Effects of ATP addition to the buffer composed of 200 mM Tris, 300 mM KCl and 12 mM MgCl_2_, pH 7.46, 7.80, 8.09 and 8.68 as measured prior to the ATP addition, and tested before and after the addition of up to 5 mM ATP. (**B**) Effects of buffer concentration on buffer capacity demonstrated as effects of ATP addition to the buffer containing either 250 mM HEPES, 375 mM KCl and 15 mM MgCl_2_ or 50 mM HEPES, 50 mM KCl and 15 mM MgCl_2_, pH 7.26 and 7.35, respectively, as measured prior to the ATP addition and tested before and after the addition of up to 5 mM ATP. (**C**) Effects of ATP addition to the buffer composed from 200 mM glycine and 12 mM MgCl_2_, pH 8.34, 8.68, 9.51 and 9.96 as measured prior to the ATP addition and tested before and after the addition of up to 5 mM ATP. (**D**) Increase in buffering capacity of the glycine buffer demonstrated as the decrease in ΔpH of 200 mM glycine and 12 mM MgCl_2_ following the increase of pH of the buffer without ATP closer to its p*K*_a_. The demonstrated pH range reflects the range of the use of glycine buffer by Salas *et al*.^5^. (**E**) The pH optimum curve of mammalian GCK reprinted with permission from Salas *et al*.^5^. Note the use of glycine buffer in the range where it is out of its buffering capacity. Republished with permission of American Society for Biochemistry and Molecular Biology, from Salas, J., Salas, M., Vinuela, E. & Sols, A.: Glucokinase of rabbit liver, J. Biol. Chem. 240, edition 1, 1965, pp. 1014–1018; permission conveyed through Copyright Clearance Center, Inc. (**F**) The pH optimum curve of human GCK generated during the course of the present study in reaction buffers containing either 200 mM Tris (pH range up to 8.5) or 200 mM glycine (pH range from 8.1).

Even relatively low concentrations of ATP (up to 5 mM) shifted pH of reaction mixtures to acidic values (Fig. 1). The ATP-induced pH shifts differed with respect to the buffering capacity, which is maximal in p*K*_a_ of the respective substance, with Tris having its p*K*_a_ at 8.07 and glycine at 9.8. Tris buffer lost its buffering capacity at pH 7.46 by adding ATP at a concentration higher than 2 mM (Fig. 1A). Similarly, glycine buffer lost its buffering capacity with the increasing difference from *pK*_a_ of glycine (Fig. 1C,D). In addition to the buffering capacity, the pH shifts differed with respect to the buffer concentration, with lower buffer concentrations leading to more prominent ATP-induced pH shifts (Fig. 1B).

To demonstrate that the ATP-induced pH shifts may severely affect outcomes of enzyme kinetics measurements, we investigated HK assays, in which high ATP concentrations are commonly used. First, we re-evaluated the pH optimum of GCK that was previously published by Salas *et al*.^5^ (Fig. 1E) based on the rabbit GCK ortholog. This is still the only reference curve of the pH optimum of mammalian GCK despite the issues reported below; the pH optimum of human GCK itself was never tested. We particularly contradicted the use of glycine buffer in the published pH range, since a pH lower than 8.0 is outside of the reliable glycine buffering range. Thus, the addition of 5 mM ATP increased the pH of the reaction mixture outside of the desired pH values (Fig. 1C). Therefore, we measured the pH optimum of human GCK in buffers that were devoid of these issues (200 mM Tris and 200 mM glycine) and reflected the pH of the whole reaction mixtures (Fig. 1F), setting the pH optimum of human GCK to pH 8.5–8.7, high above physiological intracellular pH.

**Figure 2:**
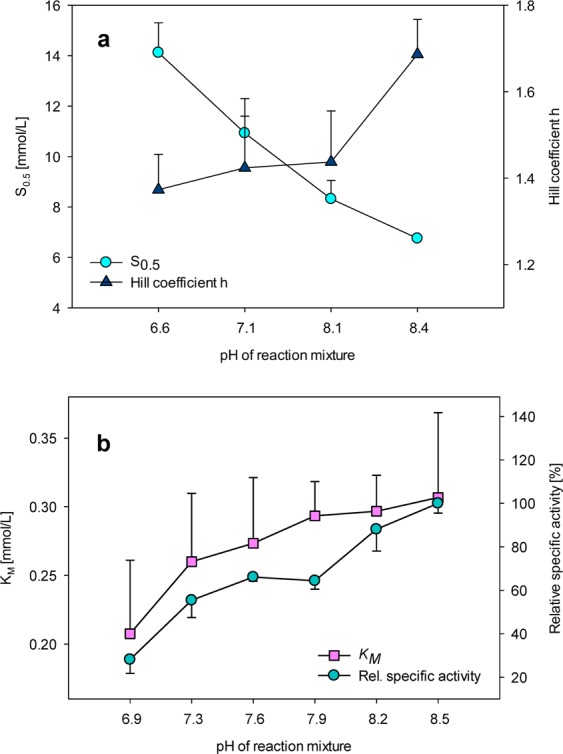
The effects of the pH on enzyme kinetics of human GCK and HK2. (**a**) *S*_*0*.*5*_ and the Hill index (*h*) of human GCK as measured in the pH range from 6.6 to 8.4. (**b**) *K*_*M*_ and relative specific activity of human HK2 as measured in the pH range from 6.9 to 8.5.

The pH optimum higher than physiological intracellular pH of adult differentiated cells is already known from some other glycolytic enzymes and is likely related to increased glycolysis in cancer cells. Similarly to the present data on GCK (Fig. 1), phosphofructokinase, a key rate-limiting glycolysis enzyme, has pH optimum at values higher than those considered physiological intracellular pH of adult differentiated cells. Moreover, the activity of phosphofructokinase drops by over two orders of magnitude when the pH decreases from 7.5 to 7.0^12,13^. Phosphofructokinase studies revealed that its inhibition by protons is allosterically modified by ATP, and the inhibitory effects are lower when lower ATP concentrations are present. The inhibitory effects of ATP at low pH can be reversed by increased concentrations of AMP^14–18^. Another glycolytic enzyme with higher than expected pH optimum is lactate dehydrogenase, which regenerates NAD^+^ for glycolysis, and which has its pH optimum at pH 7.5^19^. Consistent with the above findings, the acidosis is well known to decrease glycolytic activity both *in vitro* and *in vivo*^20–22^. Importantly, tumors have been repeatedly reported to have a higher intracellular (and lower extracellular) pH compared to normal differentiated adult cells^23,24^. The intracellular pH of cancer cells is usually between 7.3 and 7.6, whereas the normal differentiated adult cells have an intracellular pH ~7.2^24^, which applies to cancer cells from their early developmental stages^25^, is sufficient to induce dysplasia^26^, and is further facilitated during their neoplastic progression^27^. The role of the dynamics of intracellular pH remains in regulating cell fate decisions and cancer progression remains understudied^28^ as the previous biochemical studies were only recently supported by an emerging evidence of transient changes in intracellular pH during key cellular processes, including cell cycle progression^29^, migration^30,31^, or differentiation^32,33^. As a large part of previously reported data on HK kinetics is likely affected by the artifacts introduced by the addition of ATP, trusting in the buffering capacity of the respective buffers, we next focused on whether the effects of the pH changes could be generalized to all HKs. We compared the data obtained using human GCK with those obtained using HK2. The *S*_*0*.*5*_ of GCK gradually decreased by over one half with a pH increase from 6.6 to 8.4 (Fig. 2a). In contrast, the *K*_*M*_ value of HK2 gradually increased by one half with a pH increase from 6.9 to 8.5 (Fig. 2b). During the same treatment, the cooperativity of GCK remained stable or slightly increased at the highest pH interval that was tested (Fig. 2a). In contrast, the relative specific activity of HK2 was stable only in a narrow interval of pH 7.3–7.9, while it decreased substantially at pH 6.9 and increased at pH 8.2 and higher (Fig. 2b). Collectively, these data suggest that HKs do not respond to pH changes uniformly. Their pH-dependent changes in activity may be of particular importance in emerging role of HKs in dysregulated cancer metabolism^34,35^, including their involvement in pH-induced changes of cancer metabolism^36–38^.

We proved that even low concentrations of reaction components, such as ATP, may shift the pH of the reaction to undesired values and adversely influence outcomes. We suggest paying more attention to the choice of appropriate assay buffer, its concentration, buffering capacity and the influence of other substances needed for enzyme assays, since enzymes are very susceptible to pH changes. We demonstrated the problem of the sufficient choice of buffer on the pH optimum of GCK, previously measured by Salas *et al*.^5^. Due to the consideration of the pH of the whole reaction, we identified a more alkaline pH optimum of human GCK (pH 8.5–8.7) compared to the previously reported optimum of rabbit GCK (pH 7.5–8.0), which was measured under likely irreproducible conditions^5^. We must admit that the effect of ATP could be eliminated based on the use of a prebuffered ATP solution, which can be purchased or prepared homemade. Nevertheless, the use of such a solution instead of pure ATP has not been mentioned in any of the publications reporting HK assays.

The topic of pH reliability, biased assay outcomes and confusing descriptions of enzyme conditions belong to underestimated but important issues related to research integrity and reproducibility. As an example of a good practice, we would like to cite F. M. Matschinsky and colleagues^10^, who described the GCK assay as follows: “Glucokinase activity was measured spectrophotometrically using an NADP^+^ coupled assay with glucose-6-phosphate dehydrogenase as described^39^. The pH of all assays was 7.4, except for assays which assessed the inhibition of glucokinase by glucokinase regulatory protein (GKRP) where a pH of 7.1 was used.” Although Matschinsky and colleagues referred to a previous paper with regards to the method used, they completed the description of the enzyme assay with information about the pH. Notwithstanding, the common practice is rather to introduce only individual components of the reaction mixture (Table S1). Therefore, we suggest the practice of a full disclosure of reaction conditions of the experiments, including the measurement of the pH of the whole reaction mixtures.

## Supplementary information

Supplementary tables

## Data Availability

All data are available in the main text or in the supplementary materials. Figure 1E is reprinted with permission from Salas *et al*.^5^.
