# Supplementary material for: Identification of alkaline pH optimum of human glucokinase because of ATP-mediated bias correction in outcomes of enzyme assays
Source: Sci Rep. 2019 Aug 6;9:11422. doi: 10.1038/s41598-019-47883-1 (PMC6684659; doi:10.1038/s41598-019-47883-1)
Supplement: Supplementary file 1 — Supplementary tables [file 41598_2019_47883_MOESM1_ESM.pdf]

Supplementary Materials for

Title: Identification of alkaline pH optimum of human glucokinase because of ATP-mediated bias correction in outcomes of enzyme assays

Authors: Daniela Šimčíková[1], Petr Heneberg[1]*.

Affiliation: [1]Charles University, Third Faculty of Medicine, Prague, Czech Republic.

*Correspondence to: Petr Heneberg, Third Faculty of Medicine, Charles University, Ruská 87, CZ-100 00 Prague, Czech Republic, Tel: ++420 – 775 311 177, Fax: ++420 – 267 162 710, E-mail: petr.heneberg@lf3.cuni.cz.

This file includes:

Supplementary Text (Supplementary References)
Tables S1 to S2

Supplementary Text

Supplementary references

40. Salas, M., Vinuela, E. & Sols, A. Insulin-dependent synthesis of liver glucokinase in the rat. *J. Biol. Chem.* **238**, 3535–3538 (1963).

41. Redkar, V. D. & Kenkare, U. W. Solubilization, purification, and role of sulfhydryl residues. *J. Biol. Chem.* **247**, 7576–7584. (1972).

42. Magnani, M. *et al.* Pig red blood cell hexokinase: regulatory characteristics and possible physiological role. *Arch. Biochem. Biophys.* **226**, 377–387 (1983).

43. Singh, J. & Kakkar, P. Antihyperglycemic and antioxidant effect of *Berberis aristata* root extract and its role in regulating carbohydrate metabolism in diabetic rats. *J. Ethnopharmacol.* **123**, 22–26 (2009).

44. Li, X. *et al.* Iron increases liver injury through oxidative/nitrative stress in diabetic rats: Involvement of nitrotyrosination of glucokinase. *Biochimie* **94**, 2620–2627 (2012).

45. Guo, C. *et al.* Coordinated regulatory variation associated with gestational hyperglycaemia regulates expression of the novel hexokinase *HKDC1*. *Nat. Commun.* **6**, 6069 (2015).

46. Zhang, J. *et al.* c-Src phosphorylation and activation of hexokinase promotes tumorigenesis and metastasis. *Nat. Commun.* **8**, 13732 (2017).

47. Hauk, G., McKnight, J. N., Nodelman, I. M. & Bowman, G. S. The chromodomains of the Chd1 chromatin remodeler regulate DNA access to the ATPase motor. *Mol. Cell* **39**, 711–723 (2010).

48. Kiianitsa, K., Solinger, J. A. & Heyder, W. D. NADH-coupled microplate photometric assay for kinetic studies of ATP-hydrolyzing enzymes with low and high specific activities. *Anal. Biochem.* **321**, 266–271 (2003).

49. Retzlaff, M. *et al.* Asymmetric activation of the hsp90 dimer by its cochaperone aha1. *Mol. Cell* **37**, 344–354 (2010).

50. Peisley, A. *et al.* Cooperative assembly and dynamic disassembly of MDA5 filaments for viral dsRNA recognition. *Proc. Natl. Acad. Sci. U. S. A.* **108**, 21010–21015 (2011).

51. Yi, W. *et al.* Phosphofructokinase 1 glycosylation regulates cell growth and metabolism. *Science* **337**, 975–980 (2012).

Reference	Enzyme	Buffer composition	Note
Salas et al.[5], Fig. 2	Rabbit GCK	50 mM imidazole, pH 8.0; 100 mM KCl; 5 mM ethanethiol; 10 mM $MgCl_2$; 5 mM Mg ATP; 0.25 mM NADP; 0.3 U glucose-6-phosphate dehydrogenase (G6PDH); and 100 mM glucose.	Cannot be reproduced; 50 mM imidazole does not provide sufficient buffering capacity at pH 8.0.
Salas et al.[5], Fig. 2	Rabbit GCK	50 mM glycine, pH 7.7; 100 mM KCl; 5 mM ethanethiol; 10 mM $MgCl_2$; 5 mM Mg ATP; 0.25 mM NADP; 0.3 U G6PDH; and 100 mM glucose.	Cannot be used to analyze pH-dependence of enzyme kinetics; addition of 5 mM ATP to 50 mM Tris pH 7.4 leads to a decrease of the pH by several units; see Fig. 1C.
Salas et al.[40]	Rat GCK	50 mM Tris, pH 7.4; 100 mM KCl; 5 mM β-mercaptoethanol; 5 mM $MgCl_2$; 5 mM Mg ATP; 0.25 mM NADP; 0.1 U mL^{-1} G6PDH; and 100 mM glucose, 12.5 µl/mL Tris-buffered rat liver extract.	Addition of 5 mM ATP to 50 mM Tris pH 7.4 leads to a decrease of the pH by >0.5; see Fig. 1A.
Redkar & Kenkare[41]	Bovine brain HK	80 mM Tris-HCl, pH 7.4; 0.16 mM TPN; 6 mM ATP; 8 mM $MgCl_2$; 5 mM 2-mercaptoethanol; 0.4 U G6PDH; and 27 mM glucose.	Addition of 6 mM ATP to 80 mM Tris pH 7.4 leads to a decrease of the pH by >0.5; see Fig. 1A.
Magnani et al.[42]	Pig HK3	80 mM Tris-HCl, pH 7.2; 5 mM Mg ATP; 5 mM $MgCl_2$; 0.5 mM $NADP^+$; 0.1 U G6PDH; and 0.25 mM glucose.	Addition of 5 mM ATP to 80 mM Tris pH 7.2 leads to a decrease of the pH by >0.5; see Fig. 1A.
Singh & Kakkar[43]	Rat GCK	100 mM Tris, pH 7.4; 0.2 mM $NADP^+$; 5 mM ATP; 5 mM $MgCl_2$; 0.3 U G6PDH; and 0.5 mM or 100 mM glucose.	Addition of 5 mM ATP to 50 mM Tris pH 7.4 leads to a decrease of the pH by >0.5; see Fig. 1A.
Li et al.[44]	Rat GCK	50 mM HEPES; 100 mM KCl; 7.5 mM $MgCl_2$; 2.5 mM dithioerythritol; 1 mg mL^{-1} BSA; pH 7.4; 5 mM ATP; 0.5 mM NADP; 2 U G6PDH; and 0.5 mM or 100 mM glucose.	Addition of 5 mM ATP to 50 mM HEPES pH 7.4 leads to a decrease of the pH by almost one degree; see Fig. 1B.
Gui et al.[45]	Human HKDC1	50 mM HEPES, pH 7.4; 100 mM KCl; 8 mM $MgCl_2$; 5 mM ATP; 0.5 mM NADP, 1 U mL^{-1} G6PDH; 1 mM DTT; 1 g L^{-1} BSA; and 10 mM glucose; mixed 5:1 with 20 mM KH_2PO_4; 100 mM KCl; 1 mM $MgCl_2$; 1 mM EDTA; 1 mM DTT; 60 g L^{-1} glycerol, and 1 g L^{-1} BSA	Addition of 5 mM ATP to 50 mM HEPES pH 7.4 leads to a decrease of the pH by almost one degree; see Fig. 1B.
Zhang et al.[46]	Total human HK	100 mM Tris-HCl, pH 8.0; 0.5 mM EDTA; 10 mM ATP; 10 mM $MgCl_2$; 0.2 mM $NADP^+$; 30 µg of G6PDH; and 2 mM glucose.	Approximation of the data presented in Fig. 1a suggests that 10 mM ATP decreases pH of 100 mM Tris pH 8.0 by ~0.5.

Table S1.

Examples of previously published experiments on the enzyme kinetics of hexokinases that are likely affected by the ATP supplementation or, alternatively, that may have used buffered ATP but did not report it. The studies represent those that involved the addition of 5 mM ATP to the buffers at or beyond their buffering capacity, with the pH adjusted prior the ATP addition.

Assay	Reference	Buffer composition	ATP effect
ATPase assay	Hauk et al.[47]	10 mM HEPES, pH 7.8; 10 mM $MgCl_2$; 100 µM EDTA; 10% glycerol; 2.5 mM ATP; 100 mM or 50 mM KCl; 18–100 nM Chd1; 400 µg/mL NADH; 2.5 mM PEP; and 5 U of PK/LDH.	Fig. 1B
ATPase assay	Kiianitsa et al.[48]	25 mM triethanolamine acetate, pH 7.5; 13 mM $Mg(CH_3COO)_2$; 1.8 mM dithiothreitol; 5 mM ATP; 100 µg/mL BSA; 20 U mL^{-1} LDH; 3 mM PEP; and 20 U mL^{-1} PK.	N/T, likely shifts the pH out of the effective pH interval (7.3–8.3)
ATPase assay	Retzlaff et al.[49]	40 mM HEPES/KOH, pH 7.5; 20 mM KCl; 10 mM $MgCl_2$; and 5 mM ATP.	Fig. 1B
ATPase assay	Peisley et al.[50]	20 mM HEPES, pH 7.5; 150 mM NaCl; 1.5 mM $MgCl_2$; 2 mM DTT; and 2 mM ATP	Fig. 1B
Chd1 nucleosome sliding assay	Hauk et al.[47]	10 mM HEPES, pH 7.8; 10 mM $MgCl_2$; 100 µM EDTA; 10% glycerol; 5 mM ATP; and 100 mM or 50 mM KCl.	Fig. 1B
Phosphofructokinase 1 (PFK1) assay	Yi et al.[51]	50 mM Tris-HCl, pH 7.5; 100 mM KCl; 5 mM $MgCl_2$; 0.05 – 10 mM ATP; 0.2 mM NADH; 5 mM Na_2HPO_4; 0.1 mM AMP; 1 mM NH_4Cl; 5 mM fructose-6-phosphate; 5 U of triose phosphate isomerase; 1 U of aldolase; 1 U of α-glycerophosphate dehydrogenase	Fig. 1A

Table S2.

Examples of enzyme kinetics assays (other than hexokinase assays) that are likely affected by the ATP supplementation or, alternatively, that may have used buffered ATP but did not report it. The studies represent those that involved the addition of 5 mM ATP to the buffers at or beyond their buffering capacity, with the pH adjusted prior to the ATP addition.
